# PatientExploreR: an extensible application for dynamic visualization of patient clinical history from electronic health records in the OMOP common data model

**DOI:** 10.1093/bioinformatics/btz409

**Published:** 2019-06-19

**Authors:** Benjamin S Glicksberg, Boris Oskotsky, Phyllis M Thangaraj, Nicholas Giangreco, Marcus A Badgeley, Kipp W Johnson, Debajyoti Datta, Vivek A Rudrapatna, Nadav Rappoport, Mark M Shervey, Riccardo Miotto, Theodore C Goldstein, Eugenia Rutenberg, Remi Frazier, Nelson Lee, Sharat Israni, Rick Larsen, Bethany Percha, Li Li, Joel T Dudley, Nicholas P Tatonetti, Atul J Butte

**Affiliations:** 1 Bakar Computational Health Sciences Institute, University of California, San Francisco, San Francisco, CA, USA; 2 Department of Biomedical Informatics, Columbia University, New York, NY, USA; 3 Department of Systems Biology, Columbia University, New York, NY, USA; 4 Department of Medicine, Columbia University, New York, NY, USA; 5 Departments of Genomics and Data Science, Icahn Institute for Genomic Sciences and Multiscale Biology, Icahn School of Medicine at Mount Sinai, Institute of Next Generation Healthcare, New York, NY, USA; 6 Division of Gastroenterology, Department of Medicine, University of California, San Francisco, CA, USA; 7 Enterprise Information and Analytics, University of California, San Francisco, San Francisco, CA, USA; 8 Center for Data-Driven Insights and Innovation, University of California Health, Oakland, CA, USA

## Abstract

**Motivation:**

Electronic health records (EHRs) are quickly becoming omnipresent in healthcare, but interoperability issues and technical demands limit their use for biomedical and clinical research. Interactive and flexible software that interfaces directly with EHR data structured around a common data model (CDM) could accelerate more EHR-based research by making the data more accessible to researchers who lack computational expertise and/or domain knowledge.

**Results:**

We present PatientExploreR, an extensible application built on the R/Shiny framework that interfaces with a relational database of EHR data in the Observational Medical Outcomes Partnership CDM format. PatientExploreR produces patient-level interactive and dynamic reports and facilitates visualization of clinical data without any programming required. It allows researchers to easily construct and export patient cohorts from the EHR for analysis with other software. This application could enable easier exploration of patient-level data for physicians and researchers. PatientExploreR can incorporate EHR data from any institution that employs the CDM for users with approved access. The software code is free and open source under the MIT license, enabling institutions to install and users to expand and modify the application for their own purposes.

**Availability and implementation:**

PatientExploreR can be freely obtained from GitHub: https://github.com/BenGlicksberg/PatientExploreR. We provide instructions for how researchers with approved access to their institutional EHR can use this package. We also release an open sandbox server of synthesized patient data for users without EHR access to explore: http://patientexplorer.ucsf.edu.

**Supplementary information:**

Supplementary data are available at *Bioinformatics* online.

## 1 Introduction

Large-scale electronic health record (EHR) data have demonstrated the potential to completely transform the process of scientific discovery in precision medicine (Glicksberg *et al.*, 2019; Jensen *et al.*, 2012). The ‘real world data’ contained within EHRs can benefit scientists and physicians across a range of disciplines (Frankovich *et al.*, 2011). However, challenges remain that limit effective use of these data for research, including incompatible data framework between institutions as well as a lack of technical and domain expertise for researchers.

In recent years, standardized data models, such as the Observational Medical Outcomes Partnership (OMOP) common data model (CDM) (https://www.ohdsi.org/data-standardization/the-common-data-model/), developed by Observational Health Data Sciences and Informatics (OHDSI; https://www.ohdsi.org/) or the Fast Healthcare Interoperability Resources (FHIR; https://www.hl7.org/fhir/) framework have been developed and reduced interoperability issues in EHR-based research. Frameworks such as these have lowered the barrier for cross-institution collaborations and enabled the verification of new discoveries across diverse institutional settings and the replicability of key findings (Duke *et al.*, 2017; Hripcsak *et al.*, 2016; Rajkomar *et al.*, 2018; Vashisht *et al.*, 2018).

However, for many researchers the usability of EHR data is hindered by lack of programming expertise and/or familiarity with EHR database structure, even in CDMs like these that are efficiently designed. Interactive visualization applications that seamlessly interface with EHR systems could benefit such researchers by facilitating dynamic exploration and rapid extraction of patient data. Many such applications, including those on the FHIR apps platform (Mandel *et al.*, 2016), already exist (some of which even overlay statistical analyses) and have proven successful in this endeavor (Badgeley *et al.*, 2016; Krause *et al.*, 2016; Malik *et al.*, 2015; Perer *et al.*, 2015; Rind *et al.*, 2013; Soulakis *et al.*, 2015; West *et al.*, 2015; Zhang *et al.*, 2015). For example, HARVEST is a powerful but proprietary point-of-care tool that automatically synthesizes, summarizes and visualizes longitudinal patient records with a particular emphasis on data extracted from clinical notes (Hirsch *et al.*, 2015). The study authors have even demonstrated HARVEST's positive impact in direct clinical medicine. In light of interoperability goals, another system, DQ^e^-v is a database-agnostic framework for visually exploring variability in EHR data across sites and time (Estiri and Stephens, 2017). The OHDSI group has produced a substantial amount of research and open-source resources, packages, tools, applications and methodologies that enhance and facilitate OMOP CDM-based EHR research at all levels (Hripcsak *et al.*, 2015; Levine *et al.*, 2018; Schuemie *et al.*, 2018; Shaddox *et al.*, 2016). For example, Achilles (http://www.ohdsi.org/web/achilles/) (Huser *et al.*, 2016) is a dashboard resource that produces high-level, interactive cohort descriptive plots (called Reports), as well as a data quality check through its Achilles Heel. While these reports are useful in providing aggregated information about the dataset (e.g. data density trend lines, demographic breakdown, etc.), they do not allow for user interaction (i.e. browsing and filtering) and are limited in the types of plots produced. These data are primarily displayed as a treemap plot visualizing normalized sizes of different concepts based on prevalence, which then can be broken down into statistics about patients that have each clinical concept. However, individual patient-level longitudinal data (i.e. measurement values over time) cannot be viewed. ATLAS (http://www.ohdsi.org/web/atlas/) is another powerful application that allows users to browse concepts, query and define cohorts, load and visualize OMOP-formatted EHR data and even the ability to perform some predictive analyses. In the Profiles section, users can visualize multiple modalities of data per patient over time in a combined dot plot, but visualizing nuanced trends, like in actual measurement values, is not possible.

Effective visualization in particular is a challenge: utilities must carefully and selectively curate which data to display to maximize information gain without overload (Pivovarov and Elhadad, 2015). In our own work, we desired a system that would (i) allow for seamless browsing and filtering of aggregate patient data, (ii) provide a graphical representation of a single patient record, (iii) visualize longitudinal patient data, including both categorical and numeric (e.g. lab value) data in a way that was dynamic and interactive and (iv) enable data, both individual and aggregate, to be quickly and easily exported for use by other software. While the existing tools are individually powerful, no single tool provided all the functionality we needed.

To this end, we have created an open source application for EHR data in OMOP CDM that allows for easy querying and extraction of data as well as effective visualizations of patient-level data (e.g. interactive timeline visualizations and multi-domain linked graphs). Our tool, *PatientExploreR*, can be deployed on the system of any user with access to OMOP-formatted EHR data with little to no configuration. By providing a sandbox server of synthesized clinical data for exploration of our application, we hope to continue to lower the barrier of entry for researchers of all kinds to utilize EHR data in their own work.

## 2 Materials and methods

This package was made possible by the open-source packages and tools developed by the *R* and *shiny* (Chang *et al.*, 2015) communities as well as the OHDSI consortium. Due to space limitations, we describe the package structure and all application components in detail in the Supplementary Materials. There, we also detail installation and execution instructions, as well as, speed and performance information, and a strategy to deploy this app on a server environment.

### 2.1 Synthesized patient data and the public sandbox server

We have created a public sandbox server to allow individuals without access to EHR data in OMOP format to explore the visualization dashboard using synthesized clinical data. This resource is hosted at http://patientexplorer.ucsf.edu. It contains no Protected Health Information (see Supplementary Materials) and there is no registration required for use. Full details of the server setup and data sources can be found in the Supplementary Materials.

## 3 Applications

**Fig. 1.**
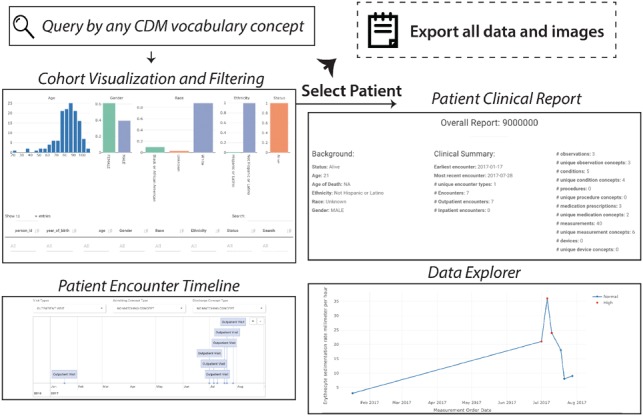
Basic functionality of *PatientExploreR* (see Supplementary Figures for more details). *PatientExploreR* allows for dynamic exploration and visualization of cohort and patient-level EHR data in OMOP format. Users can query for cohorts using combinations of any CDM vocabulary concept in any domain. Users can visualize and export outputs of this search. Once a patient is selected, a full report of all clinical concepts can be browsed and exported. Further, users can dynamically explore encounters and clinical concepts over time in both interactive numeric line and timeline plots. Further, users can interactively plot multiple modalities at once in the Multiplex Data Explorer section

*PatientExploreR* is composed of five major components: login/landing page, patient finder, overall patient report, patient encounter timeline and patient data explorer (Fig. 1). Due to space limitations, we only briefly describe the functions of each section here However, we illustrate each feature in a sample workflow for a theoretical (i.e. manually generated) patient with Ulcerative Colitis in the Supplementary Material, which hopefully demonstrates the power of patient-level EHR data visualization.

### 3.1 Landing page

To address data privacy concerns, this app can be run and maintained either by an institution (which authorizes and authenticates itself) or by individual groups that have already obtained IRB-approved access to the EHR data. Access credentials may be stored and loaded from .Renviron files. During a user’s initial login, the app generates (and saves for quicker load) a data ontology from the OMOP concept table to map all data types (Supplementary Fig. S1).

### 3.2 Patient finder

Users can identify patients using any combination of vocabulary items in the CDM (e.g. ICD-10-CM, CPT codes, RxNorm codes, etc.). This search can be performed by either ‘and’ (i.e. requires all search terms) or ‘or’ (i.e. requires any search term) operations. These criteria can be searched for directly (‘Direct’; i.e. specific ICD10CM code) or by mapping the terms to a common ontology and finding all descendants (‘Mapped’). The resulting patient list, including all demographic features, is dynamically plotted [using plotly (Sievert *et al.*, 2017)]. It can be filtered (i.e. subset) and exported to an IRB-validated machine (Supplementary Fig. S2).

### 3.3 Overall report

Once a patient is selected, summary information about his or her background and clinical history is generated alongside a full clinical data report. This report is ordered by date and may be filtered by data type (e.g. Conditions) or specific concepts (e.g. Dehydration). All data from this report can be exported for subsequent use (Supplementary Fig. S3).

### 3.4 Interactive timeline

Users can visualize the distribution (i.e. bar chart of visit types) and a timeline of clinical encounters for a given patient. These are displayed using both the *plotly* (Sievert *et al.*, 2017) and *timevis* (Attali and Almende, 2016) packages. The timeline automatically includes all patient encounters on an adjustable timescale. Selecting a single encounter from this timeline will display all information about that encounter and all clinical data that was captured during it (Supplementary Fig. S4).

### 3.5 Data explorer

Finally, the user may explore both categorical (e.g. disease diagnoses) and numeric (e.g. lab values) data in either a targeted or multiplex fashion. Categorical data can either be plotted as a timeline (as in the patient encounter timeline) or on a dot plot. Numeric data are plotted as a line or scatter plot. In the targeted mode, one data modality (i.e. disease diagnoses over time) is displayed at a time (Supplementary Fig. S5). In the multiplex mode, disparate data types (e.g. measurements and disease diagnosis events) are plotted concurrently on the same timescale, facilitating exploration of correlations among different clinical events (Supplementary Fig. S6). In the multiplex timeline mode, all data are grouped by modality and plotted across an interactive timeline (Supplementary Fig. S7).

## 4 Conclusion

EHRs contain invaluable data that need to be better utilized to inform biomedical research across a range of disciplines. The use of standardized CDM’s such as the OMOP format facilitates interoperability across institutions. However, the continued need for computational expertise combined with domain knowledge of the EHR structure to effectively use the data for research remains a significant limitation that prevents more widespread adoption for research. Quick visualization and search of EHR data breaks down barriers to entry for researchers outside these areas of expertise. As such, we have created a dynamic visualization dashboard, PatientExploreR, that is open source and freely usable by any researcher with access to OMOP-formatted EHR data. We have also verified that this application works seamlessly across three separate institutions which implement the OMOP CDM, specifically the University of California, San Francisco, Columbia University, and the Icahn School of Medicine at Mount Sinai.

Several limitations to the utility of this application must be addressed. First, not all aspects of the OMOP data are displayed in the application (e.g. Notes). Second, it would still be beneficial for users to have some familiarity of EHR-related concepts (i.e. underlying vocabularies and ontologies) in order to make most effective use of the application features. In addition to the multitude of resources that exist to explore these concepts, we direct readers to our R package, ROMOP, which in conjunction with a step-by-step tutorial (http://romop.ucsf.edu/), can be used to better understand the CDM and EHR concepts (Glicksberg *et al.*, 2019). Moreover, the public sandbox server released with this manuscript (http://patientexplorer.ucsf.edu) allows for users without data access to gain familiarity with, explore and visualize synthesized EHR data. Finally, this application will only work with data properly formatted to the OMOP CDM. The process to convert EHR data to this format is not trivial and requires a substantial amount of time and effort by a trained team. For this task, we direct users to the detailed resources provided by the OHDSI group (https://www.ohdsi.org/data-standardization/). Despite these limitations, we hope that researchers can utilize and build upon this application to facilitate more widespread adoption of the OMOP CDM. The application and all supporting materials are freely available on GitHub (https://github.com/BenGlicksberg/PatientExploreR).

## Supplementary Material

btz409_Supplementary_Data
