# Supplementary material for: PatientExploreR: an extensible application for dynamic visualization of patient clinical history from electronic health records in the OMOP common data model
Source: Bioinformatics. 2019 Jun 19;35(21):4515–8. doi: 10.1093/bioinformatics/btz409 (PMC6821222; doi:10.1093/bioinformatics/btz409)
Supplement: btz409_Supplementary_Data [file btz409_supplementary_data.zip › btz409-suppl_data/patientexplorer_bioinformatics_supplementary_materials_revision_final.docx]

**Title**

PatientExploreR: an extensible application for dynamic visualization of patient clinical history from Electronic Health Records in the OMOP Common Data Model

**Authors**

Benjamin S. Glicksberg^1^, Boris Oskotsky^1^, Phyllis M. Thangaraj^2^^, Nicholas Giangreco^2^^, Marcus A. Badgeley^3^^, Kipp W. Johnson^3^^, Debajyoti Datta^1^, Vivek Rudrapatna^1,4^, Nadav Rappoport^1^, Mark M. Shervey^3^, Riccardo Miotto^3^, Theodore C Goldstein^1^, Eugenia Rutenberg^1^, Remi Frazier^5^, Nelson Lee^5^, Sharat Israni^1^, Rick Larsen^5^, Bethany Percha^3^, Li Li^3^, Joel T. Dudley^3^, Nicholas P. Tatonetti^2^, Atul J. Butte^1,6*^

**Affiliations**

1. Bakar Computational Health Sciences Institute, University of California San Francisco, San Francisco, CA, 94158, USA.
2. Departments of Biomedical Informatics, Systems Biology, and Medicine, Columbia University, New York, New York, 10032, USA.
3. Departments of Genomics and Data Science, Icahn Institute for Genomic Sciences and Multiscale Biology, Icahn School of Medicine at Mount Sinai, Institute of Next Generation Healthcare, New York, NY 10029, USA
4. Division of Gastroenterology, Department of Medicine, University of California, San Francisco, CA, 94158, USA.
5. Enterprise Information and Analytics, University of California, San Francisco, San Francisco, CA, 94158 USA
6. Center for Data-Driven Insights and Innovation, University of California Health, Oakland, CA 94607 USA

^^^ contributed equally

**Correspondence*: atul.butte@ucsf.edu

*Package maintainer:* benjamin.glicksberg@ucsf.edu

**Supplementary Materials**

*Package installation instructions*

Users can set up PatientExploreR for use with their own institution’s EHR data by obtaining the app from https://github.com/BenGlicksberg/PatientExploreR. This can be downloaded from the website or cloned by navigating to the user’s desired local directory and typing the following command: git clone https://github.com/BenGlicksberg/PatientExploreR. Running Rscript install.R will ensure all necessary packages are installed. To run the app, type the command: R -e "shiny::runApp('app.R ')", then navigate to the IP address in an internet browser after “Listening on…”.

*Package structure and components*

*PatientExploreR* is built in *R* (version 3.4.1) using the *Shiny (Chang, et al., 2015)* (version 1.0.5) framework and directly interfaces with OMOP-formatted (version 5 or later) EHR data. The code and application are freely available on GitHub: https://github.com/BenGlicksberg/PatientExploreR, which also contains extensive documentation and installation instructions. In the front-end, the following *Shiny*-related packages are utilized: *shinyWidgets* (Perrier and Meyer, 2018), *shinyjs* (Attali, 2017)*, shinyalert* (Attali and Edwards, 2018)*,* and shinythemes (Chang, 2015). Visualizations were created using the *plotly* (Sievert, et al., 2017) and *timevis* (Attali and Almende, 2016) packages.

In its backend, *PatientExploreR* makes use of *ROMOP* (https://github.com/BenGlicksberg/ROMOP) to automatically extract and map pertinent concepts across all relevant tables (e.g., person, observation, and condition occurrence). Data processing and manipulation were facilitated by devtools (Wickham and Chang, 2016), *stringr* (Wickham, 2015), *purr* (Henry and Wickham, 2017), *data.table* (Dowle, et al., 2018)*, DT* (Xie, et al., 2015), and *dplyr* (Wickham, et al., 2015)*. Shiny-directory-input* (Pang, 2018) is also utilized but is installed with the app as instructed by the package author. Furthermore, this app retrieves EHR data by connecting to a MySQL database (utilizing the *DBI* (James, 2012) package and *RMySQL* (Ooms, et al., 2015) driver). Our application also can interface with a variety of other relational database structures, such as MicrosoftSQL Server, Oracle Server, PostgreSQL, using the OHDSI group’s *DatabaseConnector* (Scheumie and Suchard, 2018) and *DatabaseConnectorJars* (Scheumie and Suchard, 2018) packages. The syntax of all queries was translated across these platforms using the OHDSI group’s *SqlRender* (Scheumie and Suchard, 2018) package.

*Synthesized Clinical Data Source*

The Centers for Medicare and Medicaid Services (CMS) have made available a clinical dataset of synthesized patient data, DE-SynPUF (https://www.cms.gov/Research-Statistics-Data-and-Systems/Downloadable-Public-Use-Files/SynPUFs/DE_Syn_PUF.html). This dataset is open to the public with the aim of being reflective of the patient population without containing any actual Protected Health Information. The OHDSI group has converted these data into the OMOP CDM format (https://github.com/OHDSI/ETL-CMS). For this project, we downloaded all data files from the OHDSI FTP server (accessed June 17th, 2018) and created the CDM (DDL and indexes) according to their official instructions but modified for MySQL. As the CMS data does not contain any lab measurement values, we created a fake patient profile (person id 9000000) to illustrate the utility of PatientExploreR. This patient presents with a case of Ulcerative Colitis. We provide the commands to generate this patient’s data into the CMS or any OMOP database.

*Public Sandbox Server Configuration*

We have created a sandbox server open to the public to demonstrate the functionality and utility of PatientExploreR using the synthesized DE_SynPUF dataset. For space considerations, we uploaded only one million rows of each of the data files. The sandbox server is a Rshiny server running as an Elastic Compute Cloud (EC2) instance on Amazon Web Services (AWS) querying a MySQL database server (AWS Aurora MySQL). Users can interact with the visualization dashboard with the synthesized clinical data by visiting http://patientexplorer.ucsf.edu. The app itself is contained within a docker instance (https://www.docker.com/) and configured to be run via shinyproxy (https://www.shinyproxy.io/).

*Speed and Performance*

Ultimately, speed and performance of this application will depend on either the computing or server environment on which it is deployed. We were able to verify the application works as intended at three separate institutions with varying cohort sizes: UCSF (n = 991,988 ), Columbia University (n = 6,377,222), and the Icahn School of Medicine at Mount Sinai (n = 1,941,083). For the public sandbox server (server instance, n = 1,000,000 synthesized patients) and a single active user, the docker instance was able to initialize in 3 seconds. Logging in, which includes loading the data dictionary and patient demographic information, took 36 seconds. Searching for all patients with any K51 ICD-10 code (K51.*; n = 64 concepts) took 7 seconds and resulted in 138 patients. Extracting all data for a single patient of interest (person_id = 9000000) took 5 seconds (although this manually created patient only has 51 data points to load). For two simultaneous users (two dockers), the instances were able to initialize in 5 seconds and both log in in 31 seconds. Running the package on a personal computer for the institution with the most data (Columbia University), the application took approximately 3 minutes to load. To search for all patients with any K51 code, the application took 72 seconds to return 10,427 rows, scanning >140 million rows in coniditon_occurrence table.

*Generating the Example Synthesized Patient Profile*

The Supplementary File contains the MySQL commands that can be used to generate the Ulcerative Colitis (UC) patient used to illustrate this app’s functionality.

*Utilizing the App with an Example Patient*

Once logging into the PatientExploreR Sandbox Server (http://patientexplorer.ucsf.edu; see Supplementary Figure 1), the user can navigate to the Patient Finder page either using the top panel or the button on the main page. For this example, the user selects ‘Search by Criteria’ radio button, which will pull up the criteria selection options (Supplementary Figure 2). As this example focuses on UC, the user inputs the ICD-10-CM code “K51” in the Search box. To select all of these concept codes, the user clicks the Select All button above the table, which will bring all 64 items to the Selected Criteria. The user can keep the ‘or’ Search Type (allowing for patients containing any of these codes) as well as the ‘Mapped’ Search Strategy (allowing for any SNOMED concepts contained under these selected ICD-10-CM codes). The user then clicks the Search by Criteria button to begin the query. The Show Plots button can be clicked to view this cohort as well as the Export Cohort to save it to a .csv file. For this example, the user is interested in young, male UC patients and will thus restrict the Gender field to “Male” and the Age slider to 21-30. Only one patient fits this criteria, so the user selects person id 9000000 in the patient table, which will bring this id to the Selected Patient ID textbox above. The user can click the Search button to begin loading the patient data, which will extract and map all encounter and clinical concepts.

The screen is automatically switched to the Overall Report screen, indicating successful loading of patient data (Supplementary Figure 3). The demographic background of the patient, as well as a generated clinical summary, are displayed for the selected patient at the top of the page. The clinical summary contains information such as length of time in EHR and number of conditions recorded. Below is the full report table of all clinical concepts recorded for the patient (coerced into a common format of Date, Type (modality), Event (concept), and Value (if available). This report can be filtered by selecting overall modalities to include in the Data Modalities dropdown, or by specific concepts within each. The report be exported as a .csv by clicking the Export Report button for reference or further analysis. The exported report will be based on the filtered items.

To explore trends in the selected patient’s encounters in the EHR over time, the user can select the Encounter Timeline tab at the top (Supplementary Figure 4). At the top, the overall breakdown of encounter types can be viewed, specifically Visit Types, Admitting Concepts, or Discharge as frequency bar plots by selecting the corresponding radio button under Plot Encounters. For this patient, he only has Outpatient visits and No Matching Concepts for Admitting and Discharge. Real patients should have multiple types of data. To view all encounters in the timeline, we can select all Visit Types we are interested in (in this case only Outpatient Visit), which automatically generates the timevis plot. This plot is interactive: the user can click and drag left and right to scroll across time, zoom in and out, as well as clicking on certain encounters. If the user clicks on the third encounter, all information for it are automatically produced below the plot. Further, all concepts recorded during the visit are populated and segmented by domain in the table below. Selecting the Measurements tab in this table, for instance, will show all lab values recorded during this visit when UC was diagnosed, which is in the Conditions tab.

Lastly, we can explore the specific details of the patient’s clinical data in the Data Explorer tab (Supplementary Figures 5-7). There are three Data Explorer Modes that can be selected, including: Targeted (one modality at a time); Multiplex (multiple modalities at the same time in a numeric line plot); and Multiplex Timeline (multiple modalities on the same timeline visualization plot). For the Targeted mode (Supplementary Figure 5), the user can look at categorical events (e.g., Conditions, Medications) over time in a timevis plot. By selecting all conditions available in the dropdown menu, the user can see the ordering of how these conditions manifested over time. In this case, Allergic Rhinitis preceded onset of UC. Clicking on any event brings up more information on it below the plot. For numeric data types (e.g., Measurements and Observations), the trend of values over time can be seen in a line plot. By clicking on one of these domains, such as Measurements, a frequency table of concepts and the amount of values recorded is produced. The user can click on one of interest, such as C-reactive protein [Mass/volume] in Serum or Plasma, which will automatically produce a plotly line plot. Like others, this plot is completely interactive, can be saved, and identifies values outside the given reference range (e.g., high values for timepoints two and three).

In the Multiplex option (Supplementary Figure 6), multiple data concepts of both categorical and numeric types can be plotted together to consider the phenotypic landscape of the selected patient. If the user selects all Conditions, Medications, and Measurements from the respective dropdowns, a multi-sectioned plotly line plot is produced with numeric values as line plots and categorical as dot plots. Zooming in on one plot will automatically zoom the others in the identical fashion. The user can see from this plot that relevant measurements change as the patient presents with UC, such as Albumin decreasing and Erythrocyte sedimentation rate increasing. These values soon return to normal range after medication is prescribed (e.g., infliximab 100 MG Injection [Remicade]). From viewing the trend from all relevant lab values concurrently, one could infer that the medication has produced the intended result, which is less apparent from results in text format.

In the Multiplex Timeline option (Supplementary Figure 7), multiple types of clinical domain data can also be plotted concurrently in a timevis plot. Here, the user can likewise add all Conditions, Medications, and Measurements to see when they occurred in the patient clinical trajectory. All items can be clicked on, which will produce information the selected data concept above the plot.

**Supplementary Figures**

Supplementary Figure 1: App landing page. A) 1. Tabs to select for all app sections (e.g., Patient Finder); 2. Help page for step-by-step instructions; 3. About page to learn more about the app and data sources; 4. Instructions on how to download; install; and configure app for use with users’ EHR data; 5. Instructions on how to start the Sandbox Server. B) 1. Login fields to connect to EHR database; 2. User ID; 3. Password; 4. Host (for MySQL this is typically a server address but for PostgreSQL this can be server/database); 5. Database (for MySQL this is database itself but for PostgreSQL this is schema); 6. Driver type (dropdown list of available options); 7. Port for connection; 8. Save current entered credentials to .Renviron file; 9. Load credentials from .Renviron file; 10. Set directory to save/load credentials; 11. Login/logout buttons. C) 1. Click Help to get to step-by-step instructions; 2. Patient Finder can be used for cohort querying and basic plots and information. Select a certain patient for other sections; 3. Interactive report of all clinical data generated as well as an automated clinical summary (requires a patient selected); 4. Interactive timeline plot of clinical encounters as well as frequency plots (requires a patient selected); 5. The ability to explore fine grain details about patient clinical variables over time (requires a patient selected).

Supplementary Figure 2: Patient Finder section. A) 1. Search for patients either directly or by clinical criteria (mix-and-match of any type; details below). Searching for a patient will load all relevant clinical and encounter data for subsequent sections. B) 1. Can search by all concepts contained in the CDM. Can first filter by Domain; 2. Can also filter by vocabulary; 3. Can also filter by concept class; 4. Concepts can be searched for in the search box; 5. Concepts can be selected by clicking the items in the table. This shows all concepts available based on the criteria above; 6. Can browse concept table by clicking the Next and Previous buttons. C) 1. Specific codes/concepts can be searched for (e.g., ICD10CM: K51) which filters the concept table by relevant fields; 2. The Select All button will automatically select all concepts (in this case n=64) that are filtered based on criteria; 3. The None button unselects all concepts within the filtered list; 4. The criteria filtering limits the concept space by domain (can add different types). D) 1. Can filter cohort below by any demographic feature (automatically adjusts table and plots; see below for more details); 2. Table can be sorted by clicking on columns and can show different amounts of entries per page; 3. Table can be filtered by any demographic filter options by selecting subsets below column name; 4. Lists number of patients in filtered cohort; 5. Navigate the cohort table by these control buttons; 6. Can export/save cohort demographic table into a .csv file for utility in other programs; 7. Show plots of demographic features for cohort. Dynamically changed based on filter options; 8. Other sections of the app require a patient to be selected. Clicking a patient in the table populates this field and enables searching. E) 1. Hide plots; 2. Dynamic and interactive (hover-able for tooltip text) plots of demographic features for selected cohort. Automatically altered based on filter options. Can be exported by hovering over the plot and clicking the plotly button for 'Download plot as png'.

Supplementary Figure 3: Overall Report section. A) 1. Demographic background for selected patient (in this case patient id 9000000); 2. Automatically generated clinical report for selected patient. Includes information such as time in EHR, number of encounters (and by type), and clinical modality entries (and by type). B) 1. Table of all clinical concepts recorded for selected patient. Includes formatted data for all domains and organized by Date, Type (i.e., domain), Event (i.e., concept), and Value (if available; e.g. Measurement value); 2. Browse entries in table; 3. Select Data Modalities (i.e., domains) to include in report. Selections here will filter items from report table as well as filter options in #4 (i.e., will not display any items for domain if domain not selected); 4. Filter specific concepts/items per modality to include in report

Can export all clinical data for given patient in the report (only filtered items will be saved) as a .csv file for use in other programs or for further work.

Supplementary Figure 4: Encounter Timeline section. A) 1. All encounter information for selected patient (in this case patient id 9000000). Clicking radio buttons under Plot Encounters will produce bar plots breakdown for selected type (e.g., Visit Types); 2. Select items from these fields will automatically generate and produce an interactive timeline visualization built using the timevis package. B) 1. Buttons to automatically navigate timeline. Fit All Encounters will navigate the timeline to put all items on screen (note this can increase height of plot by a lot). Focus Past Year will focus the timeline to the previous year (e.g., 2017); 2. All encounter items in the timeline can be selected. Here the third Outpatient Visit is selected for the current patient. Clicking on the timeline item populates information about the encounter below; 3. All information covered in the timeline for the encounter is displayed in the Encounter Information section; 4. All clinical data recorded during the encounter is contained within a table separated by tabs of each domain; 5. These tables contain pre-selected columns pertinent to the selected domain (e.g., 'Condition Status Type' for Condition); 6. Clicking CSV or Excel buttons on each tab will allow for downloading the table in the affiliated format.

Supplementary Figure 5: Data Explorer section (Targeted). A) 1. The Data Explorer section allows users to explore trends in categorical and numeric clinical data for a selected patient (in this case patient id 9000000). There are 3 ways in which to explore: i) Targeted: one modality at a time; ii) Multiplex: multiple modalities plotted along the same x-axis (i.e., time); and iii) Multiplex Timeline: multiple modalities plotted on a grouped timeline visualization. B) 1. The Targeted explorer option plots data based on clinical modality at a time (e.g. Conditions or Measurements). For all categorical data (Conditions, Devices, Medications, and Procedures), these are plotted as an interactive timeline; 2. For the timeline there are two view types possible: Event will display each item as a single time point; Range will plot the item from start-to-end period if that information exists (note these are not always accurate); 3. Users can select items of each modality from the dropdown to include in the plot. Only concepts recorded for the selected patient are available. C) 1. Changing View Type will automatically update timeline plot; 2. Items can be selected by clicking which will show more information about the event below. Event items (i.e., without a declared end date) will be plotted as a point; 3. Events with a declared end date will be visualized as a range. D) 1. Domains that contain numeric data, specifically Measurements and Observations (although the latter is a mix), are first displayed as a frequency table with the number of recorded events for each item; 2. Items in the frequency table can be selected by clicking to automatically produce a line plot. E) 1. Select an item from the frequency table to view trends over time for that data concept ('C reactive protein [Mass/volume] in Serum or Plasma' selected in this case); 2. An interactive line plot of all data points for the selected patients in the selected data concept is automatically produced; 3. High/Low values are automatically colored and coded based on the internal range system

Hovering over each data point displays the value.

Supplementary Figure 6: Data Explorer section (Multiplex). A) 1. Multiplex mode:

display multiple types of data on the same time scale plot; 2. Categorical data can be selected in which items are displayed as a dot plot; 3. Numerical data can be selected in which terms are displayed as a line plot. B) 1. Numeric data types (Measurements and Observations) can be selected based on what was measured for the selected patient; 2. Categorical data types (all others) can be selected in the same fashion. C) 1. The interactive multiplex plot is populated with selected items. Users can zoom in by clicking and dragging a section. All other items are then zoomed in at the same scale. Double clicking returns to original scale. Plots can be downloaded by hovering over the image and selecting 'Download plot as png'; 2. All items selected above are displayed in the legend. Categorical data are as dot plots on the top.

Supplementary Figure 7: Data Explorer Section (Multiplex Timeline). A) 1. In the Multiplex Timeline Explorer mode, multiple types of data are displayed on the same time scale in a timevis plot; 2. All data types can be selected and included based on data available from the selected patient. B) 1. Categorical data can be selected like before; 2. Numeric data can be selected as well like before. C) 1. All selected items for the selected patient are displayed in a timevis plot grouped by domain. This is interactive with the same options available as before; 2. Data can be viewed as an Event or Range as before; 3. All data items can be selected by clicking; 4. Information for the selected data item are displayed above the plot

**Supplementary References**

Attali, D. shinyjs: Easily Improve the User Experience of Your Shiny Apps in Seconds. *R package version 0.9* 2017;1.

Attali, D. and Almende, B.V. timevis: Create Interactive Timeline Visualizations in R. *R package version 0.4* 2016.

Attali, D. and Edwards, T. shinyalert: Easily Create Pretty Popup Messages (Modals) in 'Shiny'. *R package version 1.0.0.9002* 2018.

Chang, W. shinythemes: Themes for Shiny. *R package version* 2015;1(1):144.

Chang, W.*, et al.* Shiny: web application framework for R. *R package version 0.11* 2015;1(4):106.

Dowle, M.*, et al.* Package ‘data. table’. 2018.

Henry, L. and Wickham, H. purrr: Functional programming tools. *R package version 0.2* 2017;2.

James, D. DBI: R Database Interface (2009). R package version 0.2. 5. In.; 2012.

Ooms, J.*, et al.* RMySQL: database interface and MySQL driver for R. *R package version 0.10* 2015;3.

Pang, W.L. shiny-directory-input: A widget for interactive selection of directories for R Shiny Applications. *R package* 2018.

Perrier, V. and Meyer, F. shinyWidgets: Custom Inputs Widgets for Shiny}. *R package version 0.4.1* 2018.

Scheumie, M. and Suchard, M. DatabaseConnector: Connecting to Various Database Platforms. *R package version 2.2.0* 2018.

Scheumie, M. and Suchard, M. DatabaseConnectorJars: JAR Dependencies for the 'DatabaseConnector' Package. *R package version 1.0.0* 2018.

Scheumie, M. and Suchard, M. SqlRender: Rendering Parameterized SQL and Translation to Dialects. *R package version 1.5.2* 2018.

Sievert, C.*, et al.* plotly: Create Interactive Web Graphics via ‘plotly. js’. R package version 4.7. 1. In.; 2017.

Wickham, H. stringr: Simple, consistent wrappers for common string operations. *R package version* 2015;1(0).

Wickham, H. and Chang, W. devtools: Tools to Make Developing R Packages Easier. R package version 1.7. 0. In.; 2016.

Wickham, H.*, et al.* dplyr: A grammar of data manipulation. *R package version 0.4* 2015;3.

Xie, Y.*, et al.* DT: a wrapper of the JavaScript library ‘DataTables. *R package version 0.1.’Available at http://CRAN. R-project. org/package= DT [Verified 1 March 2016]* 2015.
